# Can we classify ampullary tumours better? Clinical, pathological and molecular features. Results of an AGEO study

**DOI:** 10.1038/s41416-019-0415-8

**Published:** 2019-03-06

**Authors:** Geraldine Perkins, Magali Svrcek, Cecile Bouchet-Doumenq, Thibault Voron, Orianne Colussi, Clotilde Debove, Fatiha Merabtene, Sylvie Dumont, Alain Sauvanet, Pascal Hammel, Jerome Cros, Thierry André, Jean-Baptiste Bachet, Armelle Bardier, Richard Douard, Tchao Meatchi, Frederique Peschaud, Jean-Francois Emile, Isabelle Cojean-Zelek, Pierre Laurent-Puig, Julien Taieb

**Affiliations:** 1 Sorbonne Paris – Cité, Paris Descartes University, Department of Gastroenterology and GI Oncology, Georges Pompidou European Hospital, Assistance Publique-Hôpitaux de Paris, Paris, France; 2 Centre de Recherche UMR-S 1147, Médecine Personnalisée, Pharmacogénomique, Optimisation Thérapeutique, Institut National de la Santé et de la Recherche Médicale, Paris, France; 3 Sorbonne-Université, Department of Pathology, Saint-Antoine Hospital, Assistance Publique-Hôpitaux de Paris, Paris, France; 4 INSERM, UMR S 938, Sorbonne-Université, Université Pierre et Marie Curie – Paris 6, Paris, France; 5 Department of Gastrointestinal Surgery, Pitié-Salpêtrière Hospital, Assistance Publique-Hôpitaux de Paris, Paris, France; 6 Department of Gastrointestinal Surgery, Saint Antoine Hospital, Assistance Publique-Hôpitaux de Paris, Paris, France; 7 Department of Digestive Surgery, Hôpital Beaujon, Assistance Publique-Hôpitaux de Paris, Paris, France; 8 Department of Digestive Oncology, Beaujon University Hospital, Assistance Publique-Hôpitaux de Paris, Clichy, France; 9 Centre de Recherche sur l’Inflammation (CRI), INSERM UMR 1149, University of Paris Diderot, Sorbonne Paris Cité, Paris, France; 10 Department of Pathology, Beaujon University Hospital, Assistance Publique-Hôpitaux de Paris, Clichy, France; 11 Sorbonne-Université, and department of Medical Oncology, Saint Antoine Hospital, Assistance Publique-Hôpitaux de Paris, Paris, France; 12 Department of Hepato-Gastroenterology, Pitié-Salpêtrière Hospital, Assistance Publique-Hôpitaux de Paris, Paris, France; 13 Surgical Pathology Department, Pitié-Salpêtrière Hospital, Assistance Publique-Hôpitaux de Paris, Paris, France; 14 Paris Descartes University, Department of Digestive Surgery, Georges Pompidou European Hospital, Assistance Publique-Hôpitaux de Paris, Paris, France; 15 Paris Descartes University, Department of Pathology, Georges Pompidou European Hospital, Assistance Publique-Hôpitaux de Paris, Paris, France; 16 Department of Surgery and Oncology, Ambroise Paré Hospital, Assistance Publique-Hôpitaux de Paris, Boulogne-Billancourt, France; 17 Department of Pathology, Ambroise Paré Hospital, Assistance Publique-Hôpitaux de Paris, Boulogne-Billancourt, France; 18 EA4340, Biomarqueurs en Cancérologie et Onco-Hématologie, UVSQ, Paris-Saclay University, Boulogne-Billancourt, France; 19 Department of Medical Oncology, Diaconesses Hospital, Paris, France

**Keywords:** Tumour biomarkers, Predictive markers

## Abstract

**Background:**

Ampullary adenocarcinoma (AA) originates from either intestinal (INT) or pancreaticobiliary (PB) epithelium. Different prognostic factors of recurrence have been identified in previous studies.

**Methods:**

In 91 AA patients of the AGEO retrospective multicentre cohort, we evaluated the centrally reviewed morphological classification, panel markers of Ang et al. including CK7, CK20, MUC1, MUC2 and CDX2, the 50-gene panel mutational analysis, and the clinicopathological AGEO prognostic score.

**Results:**

Forty-three (47%) of the 91 tumours were Ang-INT, 29 (32%) were Ang-PB, 18 (20%) were ambiguous (Ang-AMB) and one could not be classified. Among these 90 tumours, 68.7% of INT tumours were Ang-INT and 78.2% of PB tumours were Ang-PB. MUC5AC expression was detected in 32.5% of the 86 evaluable cases. Among 71 tumours, *KRAS, TP53, APC* and *PIK3CA* were the most frequently mutated genes. The *KRAS* mutation was significantly more frequent in the PB subtype. In multivariate analysis, only AGEO prognostic score and tumour subtype were associated with relapse-free survival. Only AGEO prognostic score was associated with overall survival.

**Conclusions:**

Mutational analysis and MUC5AC expression provide no additional value in the prognostic evaluation of AA patients. Ang et al. classification and the AGEO prognostic score were confirmed as a strong prognosticator for disease recurrence.

## Background

Ampullary adenocarcinomas (AA) are rare malignant neoplasms arising from the ampulla of Vater, a dilated conduit resulting from the union of intestinal and pancreatobiliary epithelia. Histologically, AA are heterogeneous and are classified into five subgroups: the intestinal (INT) subtype; the pancreatobiliary (PB) subtype; the mixed subtype; the mucinous subtype, and the poorly differentiated subtype.^1^ Patient outcome appears to be influenced by histopathological subtype, with the INT subtype being associated with a good prognosis in most studies.^2,3^ Moreover, this classification could help to tailor appropriately the chemotherapy regimen using fluoropyrimidine-based regimens for INT subtype tumours and gemcitabine-based regimens for PB subtype tumours. On the other hand, a retrospective AGEO (Association des Gastro-Entérologues Oncologues) study determined a prognostic score based on age, general condition, tumour differentiation and TNM stage which was independent of the histopathological subtype.^4^

Recently, immunohistochemistry (IHC)-based classification of AA has been proposed to establish INT versus PB lineage in order to stratify AA into appropriate management protocols.^5–9^ Ang et al. proposed a 4-marker panel, consisting of MUC1, CK20, CDX2 and MUC2.^5^ This panel, in combination with hematoxylin and eosin (H&E) staining evaluation allowed a dichotomous classification in 92% of cases. However, correlation with clinical behaviour was not tested by Ang et al. A recent study by Xue et al. suggested that MUC5AC could be additionally helpful in stratifying these neoplasms and also a significant independent prognostic factor.^10^

Genomic sequencing of AA has led to the identification of the genetic landscapes of this tumour type.^11–14^ We can hypothesise that the molecular AA mutation distribution may differ according to histopathological subtype and potentially help to stratify AA, especially in IHC unclassified tumours.

The aim of this study was to evaluate the Ang et al. IHC classification, the MUC5AC marker, molecular mutations, and clinicopathological factors in order to identify prognostic factors in a cohort of resected AA tumours collected in the AGEO cohort.

## Patients and Methods

### Patients and clinical data

Our study population consisted of patients with AA included in the AGEO retrospective multicentre cohort, which included 150 patients from 10 gastrointestinal oncology departments in the Paris area, France, who underwent surgical resection of their tumour between 1999 and 2010.^4^ Clinical data and follow-up information were available. The AGEO prognostic score was calculated using age, general condition, tumour differentiation and TNM stage. Formalin-fixed, paraffin-embedded (FFPE) archival tissue blocks were retrieved. Acquisition of data and biological material was approved by the Ile-de-France ethics committee number 4.

### Pathology review

Representative blocks of the tumour were chosen by local pathologists and collected centrally. The morphological subtype of each tumour was reviewed after H&E staining by a single pathologist (MS) blinded to the clinical findings and local morphological subtype classification, based on their resemblance to pancreatic/biliary (H-PB) or colonic (H-INT) carcinomas.^15^ A forced-binary approach was applied to the mixed category and these tumours were placed in either the H-PB or H-INT categories: mucinous, medullary, poorly cohesive/poorly differentiated and adenosquamous carcinomas were classified as “H-other”.^10^ These cases were analysed using the American Joint Committee on Cancer TNM staging system (seventh edition).^1^

### Tissue microarrays and IHC assessment

Tissue microarrays (TMA) were constructed from 0.6-mm diameter tissue cores obtained from FFPE tumour specimens. Four core biopsies were taken for each sample, in representative areas of the tumour. H&E staining was performed on each TMA slide to confirm the presence of tumour tissue. Staining analyses for CK7, CK20, CDX2, MUC1, MUC2 as well as MUC5AC were performed using an immunostainer, Leica Bond III, following the manufacturer’s protocols.

The cases were subclassified according to the Ang et al. classification, where INT subtype “Ang-INT” was defined as having (i) positive staining for CK20 or CDX2 or MUC2 and negative staining for MUC1, or (ii) positive staining for CK20, CDX2, and MUC2 irrespective of the MUC1 staining pattern; PB subtype “Ang-PB” was defined as having positive staining for MUC1 and negative staining for CDX2 and MUC2, irrespective of CK20 staining pattern. Staining patterns that did not fit any of the above-described patterns, were defined as ambiguous “Ang-AMB”. More than 25% of staining of tumour cells was considered as positive. The cases were also evaluated for MUC5AC, with more than 20% of staining considered as positive as previously reported.^10^ The expression of MLH1, MSH2, MSH6 and PMS2 was also assessed.^16^ Samples showing loss of protein expression on TMA were also tested on whole-section slides. The clone, source, and dilution for each marker are shown in Supplementary Table 1.

### *ERBB2* analysis

The expression of *ERBB2* was investigated on whole-section slides by IHC using the A0485 antibody (DAKO, 1:500 dilution) and graded according to breast cancer guidelines.^17^ In the case of HER2 protein over-expression (IHC score 2+ and 3+), a second test was performed by fluorescent in situ hybridisation (FISH) using the pharmDxTM test kit (Dako Denmark A/S, Glostrup, Denmark), for which HER2 protein expression is amplified as the HER2/CEP17 ratio ≥ 2.

### Molecular genotyping

Genomic DNA was extracted from punch biopsies of FFPE tumour blocks. Using a 1.0-mm punch, all FFPE tissues were sampled from representative areas that contained more than 50% of tumour cells. The same FFPE blocks were used for DNA extractions and for IHC analyses. Genomic DNA was extracted using the QIAamp FFPE DNA kit (Qiagen), according to the manufacturer’s instructions. The quantity of DNA was measured using a Qubit® 2.0 fluorometer. Molecular analysis was based on next-generation sequencing, using Ion AmpliSeq Cancer Hotspot Panel V2 (Life Technologies-Thermo Fisher Scientific), following the manufacturer’s recommendations. The list of the 50 genes targeted by this panel is shown in Supplementary Table 2. Sequencing libraries were prepared, processed and sequenced on an Ion PGM^TM^ System using an Ion 318^TM^ Chip Kit v2. Annotation was done using Ion reporter.

### Statistical analysis

All qualitative variables were compared using the chi-square test; all quantitative variables were compared using the *t*-test. Survival in the different groups was compared using the logrank test. Multivariate survival analyses were performed using the variable showing a prognostic impact in univariate analysis. A p-value of less than 0.05 was taken as significant. The statistical analysis was performed using R software and the survival package.

## Results

### Clinicopathological features of patients

**Fig. 1:**
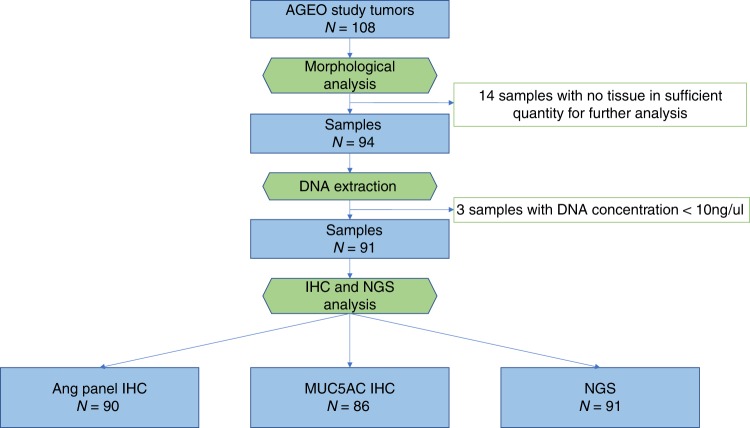
Study flowchart. IHC = immunohistochemistry, NGS = next-generation sequencing

Of 108 tumour blocks from the 150 patients included in the AGEO retrospective study, 91 were available for next-generation sequencing and morphological analysis (Fig. 1). The characteristics of these 91 patients are presented in Supplementary Table 3. A total of 21 tumours were reclassified in a subtype different from that of the local morphological classification, following central pathological review (data not shown). Half the patients (50.7%) presented an advanced stage at diagnosis (IIb or III). All patients were free of metastases at the time of surgery. The median follow-up was 35.3 months. The AGEO prognostic score was calculated and 12 (13.2%), 53 (58.2%), 26 (28.6%) patients were identified as high, intermediate and low risk, respectively. Among the 85 tumours evaluable for microsatellite analysis, 2 were MMR deficient (dMMR), exhibiting loss of MLH1 expression. Among the 84 tumours available for HER expression, 1 was positive (3+), and 4 were equivocal (2+). *ERBB2* amplification was identified only in the tumour with HER2 3+ expression.

### Ang et al. panel and MUC5AC results

Using the Ang et al. IHC typing system, 43 (47%) of the 91 tumours were classified as Ang-INT, 29 (32%) were Ang-PB, 18 (20%) were IHC ambiguous (Ang-AMB) and one could not be classified (1%), due to lack of available tissue. Therefore, 90 tumours were available for comparison between morphological classification and Ang et al. typing. After central review, 37 of the 54 H-INT tumours (68.5%) were classified as Ang-INT and 18 of the 23 H-PB tumours (78.2%) as Ang-PB. Few cross-overs between INT and PB subtypes in the two assessments were found. One H-PB tumour was found as Ang-INT (1%) and 4 H-INT tumours were assessed as Ang-PB (4.4%). Altogether, these figures give a Kappa coefficient between the morphological and IHC classification of 0.815 (95% CI [0.66–0.97]). Moreover, 17 cases of H-INT or H-PB were finally classified in the Ang-AMB group (Table 1). MUC5AC expression was detected in 32.5% of the 86 evaluable cases, 25.9% of H-INT and 47.6% of H-PB.

**Table 1:** Correlation between morphological classification and the Ang et al. classification

	Morphological typing			
	H-INT	H-other	H-PB	total
Ang typing				
Ang-INT	37	5	1	43
Ang-AMB	13	1	4	18
Ang-PB	4	7	18	29
total	54	13	23	90

### Gene alterations detected by sequencing

**Fig. 2:**
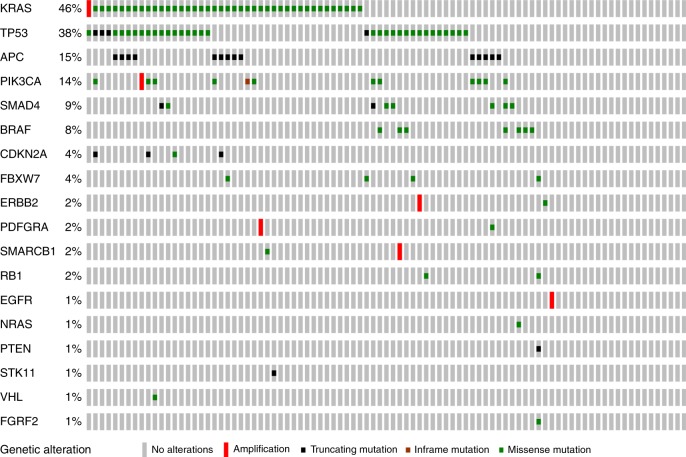
Representation of gene alterations in AA

No alteration (mutation or amplification) was detected in 20 of the 91 tumours (22%). A total of 145 alterations (138 mutations and 7 amplifications) were identified in 71 tumours. *KRAS, TP53, APC, PIK3CA, SMAD4, BRAF*, *CDKN2A, FBXW7* mutations were detected in 41, 35, 14, 12, 8, 7, 4 and 4 cases, respectively (Fig. 2). Details of mutations are listed in Supplementary Table 4. Mutations in codon 12 of *KRAS* were detected in 35 (85.4%) of 41 tumours harbouring *KRAS* mutations. The 3 most frequent mutations in codon 12 were G12D (31.7%), G12V (17.1%) and G12R (17.1%). *BRAF* and *KRAS* are mutually exclusive, and 1 tumour harboured a double mutation in the *BRAF* and *NRAS* genes. All *BRAF* mutations occurred in the non-V600 codon. *ERBB2* amplification was detected by sequencing in 1 tumour with an intestinal subtype, and this was confirmed by IHC staining 3+ and also by FISH. *EGFR* amplification was detected in 2 tumours with the intestinal subtype. Amplification of *KRAS, PIK3CA, SMARCB* and *PDFGRA* was detected in 1 poorly differentiated carcinoma and in 3 INT-type tumours, respectively.

Supplementary figure 1 summarizes IHC and genotyping results. All sequencing data of the 91 tumours are included in supplementary Table 5.

### Clinicopathological correlation with tumour morphologic subtypes, IHC subtypes and gene mutations

**Fig. 3:**
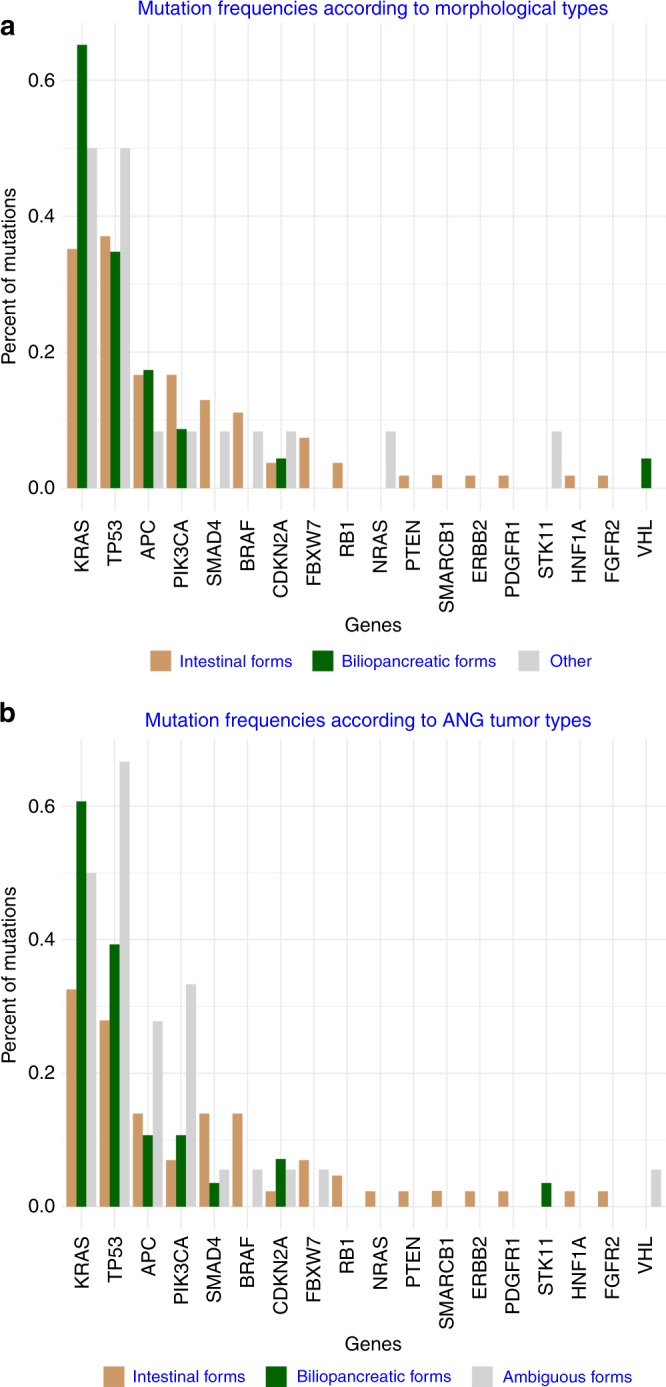
**a** Mutation frequencies according to morphological classification. **b** Mutation frequencies according to the IHC classification of Ang et al.

The distributions of the different gene mutation frequencies according to tumour type determined by morphological or Ang et al. IHC subtypes are depicted in Fig. 3a, b. According to these 2 classifications, the *KRAS* mutation was significantly more frequent in the PB subtype whatever the method used to classify tumours. *KRAS* mutations were present in 65.2% of H-PB versus 36.4% of H-INT tumours (*p* < 0.04). Similar results were observed for the IHC classification: 60.7% of the Ang-PB tumours had *KRAS*, mutations versus 32.5% of the Ang-INT tumours (*p* < 0.02). No other significant differences in terms of gene mutation frequency were observed between the 2 tumour types. Within *KRAS* exon 2, the distribution of mutations was not significantly different according to tumour type assuming low numbers (Supplementary Figure 2).

### Univariate and multivariate analysis

In univariate analysis, the mutational status of the different genes tested, as well as MUC5AC expression, were not associated with relapse-free survival (RFS) or overall survival (OS) (Table 2). Only *KRAS, TP53, APC* and *PIK3CA* were included in uni- or multivariate statistical analyses, all other mutations being too rare (1–8% of patients). Furthermore, no specific impact of the different *KRAS* exon 2 mutations on disease outcome was observed. The only significant variables associated with poor RFS in univariate analyses were the AGEO prognostic score as previously described^4^ and the tumour subtype, whether determined by morphology or IHC. Patients with the PB subtype had a poorer prognosis. In multivariate analysis, both tumour subtype and the prognostic score were associated with poor RFS (Table 2). In terms of OS, the only prognostic factor in multivariate analysis was the AGEO prognostic score (HR:22.5 95% CI 2.1–241 *p* = 0.01 for high risk and HR: 9.4 95% CI 1.2–74 *p* = 0.04 as compared to low risk, respectively).

**Table 2:** Univariate and multivariate analyses for RFS

Univariate analysis			
	HR	95% CI	*P* value
AGEO Prognostic score			
low	1		
intermediate	4.0	1.15–13.7	0.03*
high	20.5	4.45–94.7	0.000107
Morphological classification			
H-INT	1		
H-other	2.05	0.6–6.7	0.23
H-PB	3.75	1.6–9.45	<0.003
Ang et al. IHC classification			
Ang-INT	1		
Ang-AMB	0.63	0.13–3	0.56
Ang-PB	3.6	1.5–8.6	0.00467
MUC5A Expression			
Negative	1		
Positive	0.96	0.41–2.6	0.9
*KRAS* Mutation			
NM	1		
M	1.2	0.5–2.8	0,64
*TP53* Mutation			
NM	1		
M	1.1	0.5–2.6	0.8
*APC* mutation			
NM	1		
M	1.55	0.6–4.2	0.4
*PIK3CA* mutation			
NM	1		
M	0.5	0.1–2.2	0.4

Multivariate analysis according to morphological classification			
	HR	95% CI	*P* value
AGEO Prognostic score			
low	1		
intermediate	5.1	1.4–18.1	0.01
high	16.8	3.5–81.4	0.0004
Morphological classification			
H-INT	1		
H-other	2.3	0.7–7.6	0.16
H-PB	4.0	1.5–10.6	0.005

Multivariate analysis according to the IHC classification of Ang et al.			
	HR	95% CI	*P* value
Prognostic score			
low	1		
intermediate	3.8	1.07–13.3	0.04
high	18.5	3.8–90.4	0.0003
IHC classification of Ang et al.			
Ang-INT			
Ang-AMB	0.46	0.1–2.2	0.33
Ang-PB	2.7	1.1–6.7	0.03

## Discussion

AA is a rare disease whose classification and prognosis are not well established. Several tools have been investigated using clinicopathological score, morphology, and IHC classification. We sought to integrate some of these with molecular assessment, in a retrospective cohort of nearly 100 tumours.

We used tumours from the AGEO study that previously established a clinicopathological score. Compared to the original study, the distribution of the AGEO prognostic groups was slightly different, with fewer high-risk patients, probably because of sampling issues. In a recent study at the Mayo Clinic in a cohort of 121 AA patients, disease stage and ECOG were confirmed as independent prognostic factors.^18^

In the present work, the morphological subtype of each tumour was centrally reviewed by a single pathologist, unlike in previous publications. A high number of tumours were reclassified, highlighting the difficulty of classifying AA even in expert centres. We believe that the central pathology review is a strength of our study and guarantees a relatively uniform classification of our cases. However, as expected, the INT type was found to have a better prognosis.

Regarding the Ang et al. classification, we found very similar results in terms of classification of tumour subtypes, with 79% of cases labelled either Ang-INT or Ang-PB tumours, compared with 82% by Ang et al.^5^ Besides, when focusing on the “other” morphological group, only one out of 13 tumours remained ambiguous after Ang et al. typing, showing that this classification is mainly useful in tumours that are difficult to classify by morphology. Interestingly, in our study both H-PB and Ang-PB subtypes were independent factors of poor prognosis. In the study by Xue et al., only morphological classification stratified prognosis, with the Ang et al. panel approaching but not reaching significance.^10^ This discrepancy may be due to differences in terms of cohort populations, with more Ang-INT tumours in our study than in the study by Xue et al. (47% vs 35%, respectively).

MUC5AC is originally a gastric marker which is linked to worse prognosis in different tumour types, such as duodenal adenocarcinoma, cholangiocarcinoma and lung adenocarcinoma.^19–21^ Recently, it has been described as a strong prognostic factor for AA.^10^ In the current cohort, the prognostic role of MUC5A was not observed. We assessed MUC5AC staining by using TMA, unlike Xue et al. who used IHC analysis in full sections of the tumour. However, whole-section slide staining was performed in the case of TMA negative cases.

Regarding molecular classification, we found that mutations of the WNT pathway are more frequent in the INT subtype, and mutations of *KRAS* and *TP53* signalling are more frequent in the PB subtype. These trends were not significant owing to the limited size of our cohort. Our findings are similar to those of two recent studies investigating molecular profiles of AA using whole exome sequencing (WES),^11,12^ but there are minor discrepancies. When focusing on pathological subtypes, these two WES studies reported higher mutation rates of *TP53* and *CDKN2A* in the PB subtype, and higher mutation rates of *TP53* and *APC* in the INT subtype, compared to our data. These differences can be explained by the rarity of the disease, the sample size in the different cohorts that are small, and the unbalanced proportion of INT subtype and PB subtype within the cohorts (47%/29%, 34%/51 and 54%/38%, in AGEO study, Gingras et al. and Yashida et al., respectively), making difficult to compare them directly. Furthermore, the IHC method to classify INT and PB subtype were different in the 2 studies, using either Ang et al. or Chang et al. classifications respectively. According to the TCGA database, similarities of molecular profiles in terms of gene mutation frequency do exist between INT AA and colorectal adenocarcinoma, and between PB AA and pancreatic adenocarcinoma, respectively.^22,23^ This is also noted regarding *KRAS* exon 2 mutation distribution (Supplementary Figure 2). Interestingly, we found a very similar mutational profile between INT AA and our previously published small bowel cancer series, excepting the rarity of the *ERBB2* mutation in AA.^24^ Regarding the prognostic value of the different gene mutations, none was associated with survival. This finding differs from the results of a recent meta-analysis that reported a significant correlation in AA between the *KRAS* mutation and a worse RFS, but no correlation with OS.^25^ Moreover, several studies showed discrepant results regarding the prognostic role of *KRAS* mutation for RFS or OS.^13,14,26–30^ In one of them, whereas *KRAS* mutation was not associated with prognosis, patients with specific *KRAS G12D* mutation had a shorter survival compared to *KRAS non-G12D* mutation patients and *KRAS*-wild type patients.^29^ Furthermore, *KRAS* mutation appears as an early event in AA pathogenesis, as it has been reported present in 20% of adenomas and 80% of AA in a series of endoscopic papillectomy samples.^27^ Despite their poor prognosis, the finding of 30/71 *RAS* wild type, 2dMMR and one *ERBB2* amplified tumours, suggest that some patients should be eligible for targeted therapies in case of disease recurrence.

The main limitations of our work are a possible bias due to its retrospective nature and its statistical power which is limited, because of the rarity of AA.

To our knowledge, ours is the first report of a wide comprehensive assessment of molecular, IHC and clinicopathological factors for better prognostication of AA. Our findings suggest that although molecular profiling adds nothing to clinicopathological variables, IHC classification is an independent prognostic factor and should be used in daily practice to classify patients. The role of MUC5AC as a prognosticator remains to be evaluated in a large prospective series. Even when integrating the morphological classification or the IHC classification, the AGEO clinicopathological score remains highly prognostic.

## Supplementary information

Supplementary Figure 1

Supplementary Figure 2

Supplementary Table 1

supplementary Table 2

Supplementary Table 3

Supplementary Table 4

Supplementary Table 5

## Data Availability

Data and materials are available on request.
