# Supplementary material for: Can we classify ampullary tumours better? Clinical, pathological and molecular features. Results of an AGEO study
Source: Br J Cancer. 2019 Mar 6;120(7):697–702. doi: 10.1038/s41416-019-0415-8 (PMC6462032; doi:10.1038/s41416-019-0415-8)
Supplement: Supplementary file 1 — Supplementary Figure 1 [file 41416_2019_415_MOESM1_ESM.pptx]

## Slide 1
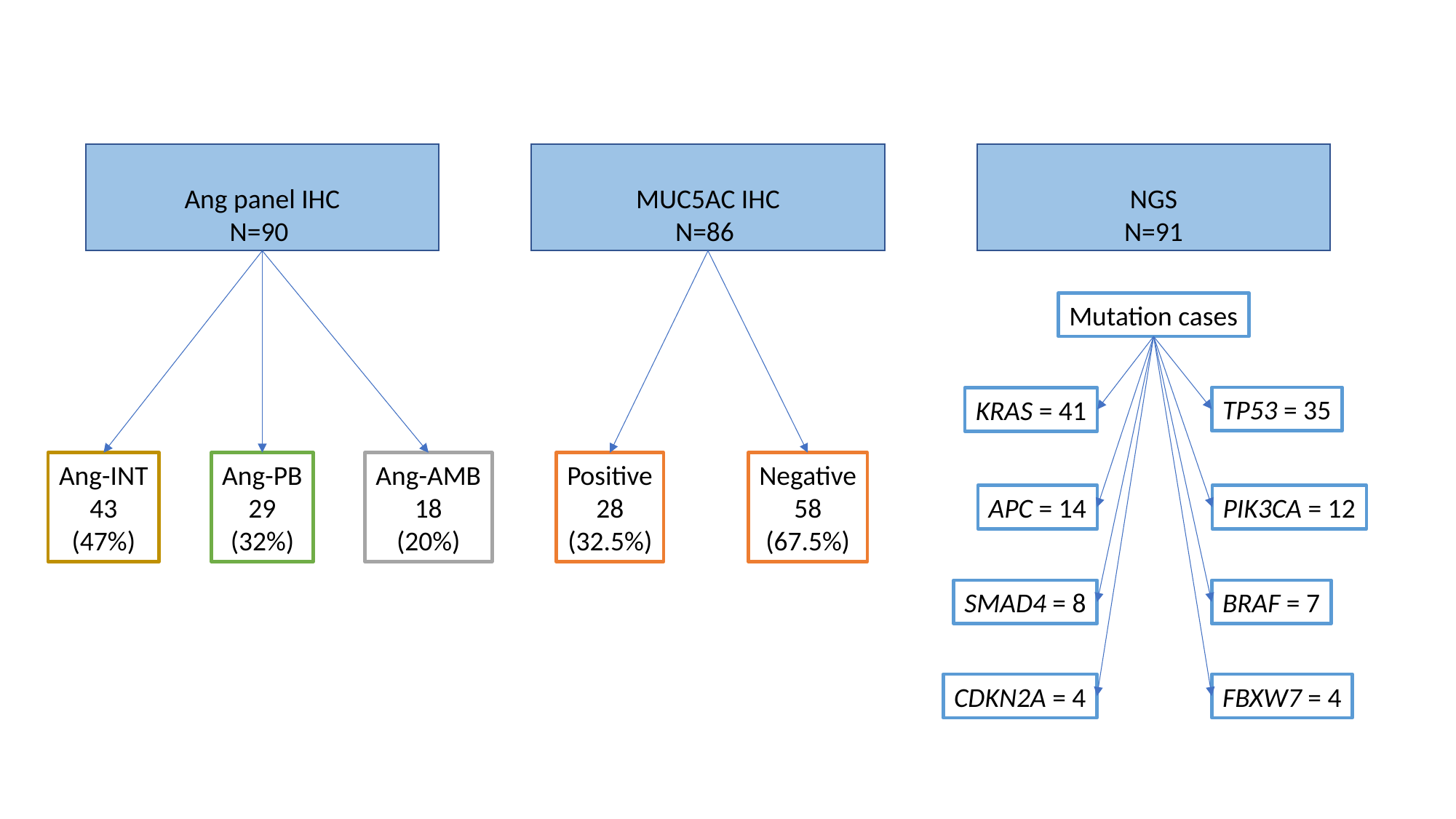

Ang panel IHC
N=90
MUC5AC IHC
N=86
NGS
N=91
Mutation cases
TP53 = 35
KRAS = 41
Ang-INT
43
(47%)
Ang-PB
29
(32%)
Ang-AMB
18
(20%)
Positive
28
(32.5%)
Negative
58
(67.5%)
APC = 14
PIK3CA = 12
SMAD4 = 8
BRAF = 7
CDKN2A = 4
FBXW7 = 4
