# Supplementary figures and images for: Can we classify ampullary tumours better? Clinical, pathological and molecular features. Results of an AGEO study
Source: Br J Cancer. 2019 Mar 6;120(7):697–702. doi: 10.1038/s41416-019-0415-8 (PMC6462032; doi:10.1038/s41416-019-0415-8)

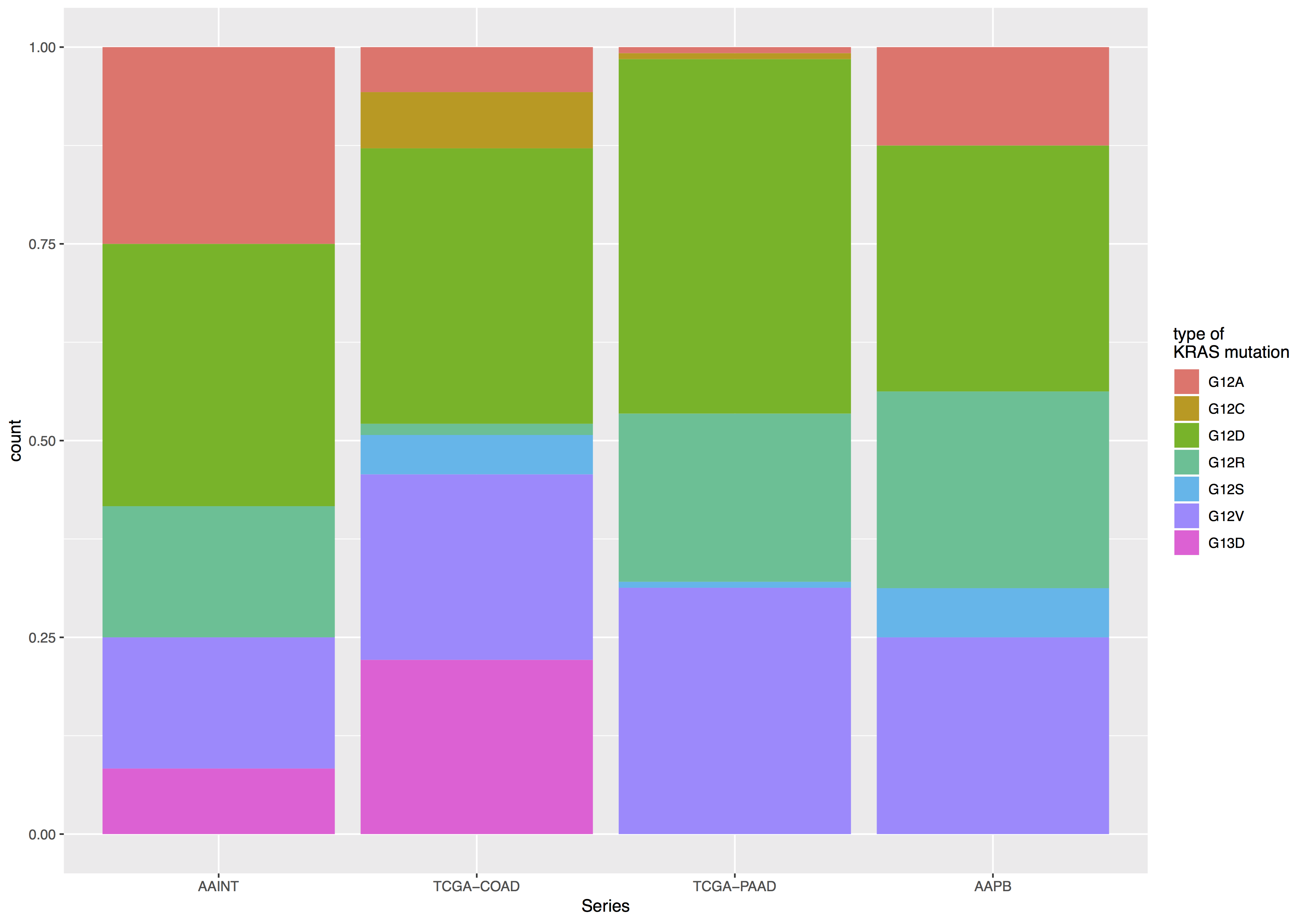

Supplement: Supplementary file 2 — Supplementary Figure 2 [file 41416_2019_415_MOESM2_ESM.png]
