# Supplementary material for: Can we classify ampullary tumours better? Clinical, pathological and molecular features. Results of an AGEO study
Source: Br J Cancer. 2019 Mar 6;120(7):697–702. doi: 10.1038/s41416-019-0415-8 (PMC6462032; doi:10.1038/s41416-019-0415-8)
Supplement: Supplementary file 3 — Supplementary Table 1 [file 41416_2019_415_MOESM3_ESM.docx]

Supplementary Table 1: Characteristics of antibodies

| Antibody | Clone | Source | Dilution |
| --- | --- | --- | --- |
| CK7 | OV-TL | DAKO | 1/50 |
| CK20 | IT-KS 20.8 | DAKO | 1/150 |
| MUC1 | Ma 695 | Novocastra | 1/200 |
| MUC2 | Ccp58 | Leica | Pre-diluted |
| MUC5AC | CLH2 | Novocastra | 1/100 |
| CDX2 | EPR2764Y | Abcam | 1/6000 |
| MLH1 | G168-728 | PharMingen | 1/70 |
| MSH2 | FE11 | Calbiochem | 1/100 |
| MSH6 | 44 | Becton Dickinson | 1/100 |
| PMS2 | A16-4 | BD PharMingen | 1/150 |
