# Supplementary material for: Can we classify ampullary tumours better? Clinical, pathological and molecular features. Results of an AGEO study
Source: Br J Cancer. 2019 Mar 6;120(7):697–702. doi: 10.1038/s41416-019-0415-8 (PMC6462032; doi:10.1038/s41416-019-0415-8)
Supplement: Supplementary file 4 — supplementary Table 2 [file 41416_2019_415_MOESM4_ESM.docx]

Supplementary Table 2: list of the 50 genes targeted by Ion AmpliSeq Cancer Hotspot Panel V2

| *ABL1* | *EGFR* | *GNAS* | *KRAS* | *PTPN11* |
| --- | --- | --- | --- | --- |
| *AKT1* | *ERBB2* | *GNAQ* | *MET* | *RB1* |
| *ALK* | *ERBB4* | *HNF1A* | *MLH1* | *RET* |
| *APC* | *EZH2* | *HRAS* | *MPL* | *SMAD4* |
| *ATM* | *FBXW7* | *IDH1* | *NOTCH1* | *SMARCB1* |
| *BRAF* | *FGFR1* | *JAK2* | *NPM1* | *SMO* |
| *CDH1* | *FGFR2* | *JAK3* | *NRAS* | *SRC* |
| *CDKN2A* | *FGFR3* | *IDH2* | *PDGFRA* | *STK11* |
| *CSF1R* | *FLT3* | *KDR* | *PIK3CA* | *TP53* |
| *CTNNB1* | *GNA11* | *KIT* | *PTEN* | *VHL* |
