# Supplementary material for: Can we classify ampullary tumours better? Clinical, pathological and molecular features. Results of an AGEO study
Source: Br J Cancer. 2019 Mar 6;120(7):697–702. doi: 10.1038/s41416-019-0415-8 (PMC6462032; doi:10.1038/s41416-019-0415-8)
Supplement: Supplementary file 5 — Supplementary Table 3 [file 41416_2019_415_MOESM5_ESM.docx]

Supplementary Table 3: Clinicopathological baseline characteristics

|  | N (%) |
| --- | --- |
| Age Median (N) | 64.8 years |
| Sex | 91 |
| Male | 51 (56%) |
| Female | 40 (44%) |
| WHO | 66 |
| 0 | 28 (42.4%) |
| 1 | 33 (50%) |
| 2 | 5 (7.6%) |
| Stage | 77 |
| Ia | 11 (14.2%) |
| Ib | 19 (24.7%) |
| IIa | 8 (10.4%) |
| IIb | 36 (46.8%) |
| III | 3 (3.9%) |
| Histological Type | 91 |
| H-INT | 55 (60.4%) |
| H-PB | 23 (25.3%) |
| H-other | 13 (14.3%) |
