# Supplementary material for: Can we classify ampullary tumours better? Clinical, pathological and molecular features. Results of an AGEO study
Source: Br J Cancer. 2019 Mar 6;120(7):697–702. doi: 10.1038/s41416-019-0415-8 (PMC6462032; doi:10.1038/s41416-019-0415-8)
Supplement: Supplementary file 6 — Supplementary Table 4 [file 41416_2019_415_MOESM6_ESM.docx]

Supplementary Table 4: list of gene mutations identified in the 91 tumors

| gene | mutation |
| --- | --- |
| *APC* | p.A1553fs |
| *APC* | p.C1578* |
| *APC* | p.E1309fs |
| *APC* | p.E1374fs |
| *APC* | p.E1451* |
| *APC* | p.E1547fs |
| *APC* | p.E1550* |
| *APC* | p.E1552* |
| *APC* | p.E1554fs |
| *APC* | p.E1576* |
| *APC* | p.K1437* |
| *APC* | p.K1462fs |
| *APC* | p.Q1444* |
| *APC* | p.R1114* |
| *BRAF* | p.D594G |
| *BRAF* | p.D594N |
| *BRAF* | p.G469A |
| *BRAF* | p.H608Y |
| *BRAF* | p.K601E |
| *BRAF* | p.N581I |
| *BRAF* | p.N581S |
| *CDKN2A* | p.D108Y |
| *CDKN2A* | p.G55fs |
| *CDKN2A* | p.R80* |
| *CDKN2A* | p.V51fs |
| *ERBB2* | p.D769Y |
| *FBXW7* | p.R441Q |
| *FBXW7* | p.R465C |
| *FBXW7* | p.R465H |
| *FBXW7* | p.S582L |
| *FGFR2* | p.N549K |
| *HNF1A* | p.T196fs |
| *HNF1A* | p.T196A |
| *KRAS* | p.G12A |
| *KRAS* | p.G12C |
| *KRAS* | p.G12D |
| *KRAS* | p.G12R |
| *KRAS* | p.G12S |
| *KRAS* | p.G12V |
| *KRAS* | p.G13D |
| *KRAS* | p.L19F |
| *KRAS* | p.Q61H |
| *KRAS* | p.Q61K |
| *NRAS* | p.G12R |
| *PDGFR1A* | p.A821S |
| *PIK3CA* | p.A400V |
| *PIK3CA* | p.E542K |
| *PIK3CA* | p.E545K |
| *PIK3CA* | p.E81K |
| *PIK3CA* | p.I391M |
| *PIK3CA* | p.L113del |
| *PIK3CA* | p.Q546K |
| *PIK3CA* | p.R108H |
| *PTEN* | p.K62fs |
| *RB1* | p.R334T |
| *RB1* | p.R579Q |
| *SMARCB1* | p.R377H |
| *SMAD4* | p.C363Y |
| *SMAD4* | p.D355G |
| *SMAD4* | p.G352E |
| *SMAD4* | p.G365D |
| *SMAD4* | p.G386V |
| *SMAD4* | p.G508fs |
| *SMAD4* | p.Q248* |
| *SMAD4* | p.R361H |
| *STK11* | p.L55fs |
| *TP53* | p.C135W |
| *TP53* | p.C135Y |
| *TP53* | p.C176* |
| *TP53* | p.C176Y |
| *TP53* | p.E180* |
| *TP53* | p.E285K |
| *TP53* | p.E286K |
| *TP53* | p.G245D |
| *TP53* | p.I162fs |
| *TP53* | p.L350P |
| *TP53* | p.M160V |
| *TP53* | p.M237I |
| *TP53* | p.P151S |
| *TP53* | p.P152L |
| *TP53* | p.Q136fs |
| *TP53* | p.R156P |
| *TP53* | p.R175H |
| *TP53* | p.R213* |
| *TP53* | p.R273C |
| *TP53* | p.R280I |
| *TP53* | p.R282W |
| *TP53* | p.R282W |
| *TP53* | p.R306* |
| *TP53* | p.R337L |
| *TP53* | p.R342* |
| *TP53* | p.S215R |
| *TP53* | p.V216M |
| *TP53* | p.Y163C |
| *TP53* | p.Y220C |
| *TP53* | p.Y234C |
| *TP53* | p.Y234N |
| *VHL* | p.R167W |
